# EEG-Based Brain Functional Connectivity in First-Episode Schizophrenia Patients, Ultra-High-Risk Individuals, and Healthy Controls During P50 Suppression

**DOI:** 10.3389/fnhum.2019.00379

**Published:** 2019-11-14

**Authors:** Qi Chang, Meijun Liu, Qing Tian, Hua Wang, Yu Luo, Jicong Zhang, Chuanyue Wang

**Affiliations:** ^1^School of Biological Science and Medical Engineering, Beihang University, Beijing, China; ^2^Beijing Advanced Innovation Centre for Biomedical Engineering, Beihang University, Beijing, China; ^3^Beijing Advanced Innovation Center for Big Data-Based Precision Medicine, Beihang University, Beijing, China; ^4^The National Clinical Research Center for Mental Disorders and Beijing Key Laboratory of Mental Disorders, Beijing Anding Hospital, Capital Medical University, Beijing, China; ^5^Advanced Innovation Center for Human Brain Protection, Capital Medical University, Beijing, China; ^6^Hefei Innovation Research Institute, Beihang University, Hefei, China; ^7^School of Biomedical Engineering, Anhui Medical University, Hefei, China

**Keywords:** EEG, ERP, functional brain connectivity, P50, first-episode, schizophrenia, ultra-high risk, classification

## Abstract

Dysfunctional processing of auditory sensory gating has generally been found in schizophrenic patients and ultra-high-risk (UHR) individuals. The aim of the study was to investigate the differences of functional interaction between brain regions and performance during the P50 sensory gating in UHR group compared with those in first-episode schizophrenia patients (FESZ) and healthy controls (HC) groups. The study included 128-channel scalp Electroencephalogram (EEG) recordings during the P50 auditory paradigm for 35 unmedicated FESZ, 30 drug-free UHR, and 40 HC. Cortical sources of scalp electrical activity were recomputed using exact low-resolution electromagnetic tomography (eLORETA), and functional brain networks were built at the source level and compared between the groups (FESZ, UHR, HC). A classifier using decision tree was designed for differentiating the three groups, which uses demographic characteristics, MATRICS Consensus Cognitive Battery parameters, behavioral features in P50 paradigm, and the measures of functional brain networks based on graph theory during P50 sensory gating. The results showed that very few brain connectivities were significantly different between FESZ and UHR groups during P50 sensory gating, and that a large number of brain connectivities were significantly different between FESZ and HC groups and between UHR and HC groups. Furthermore, the FESZ group had a stronger connection in the right superior frontal gyrus and right insula than the HC group. And the UHR group had an enhanced connection in the paracentral lobule and the middle temporal gyrus compared with the HC group. Moreover, comparison of classification analysis results showed that brain network metrics during P50 sensory gating can improve the accuracy of the classification for FESZ, UHR and HC groups. Our findings provide insight into the mechanisms of P50 suppression in schizophrenia and could potentially improve the performance of early identification and diagnosis of schizophrenia for the earliest intervention.

## Introduction

Sensory gating, defined as the ability of the brain to separate important from irrelevant sensory stimuli, is one of the early stages of information processing and cognition (Hall et al., 2010). In assessing sensory gating, a mature neurophysiological component is P50 event-related potentials, a positive wave arising around 50 ms after stimulus presentation. The P50 is evaluated using a paired-click paradigm with an interval of 500 ms. The P50 component of the first stimulus (S1) represents the individual’s response to normal auditory stimuli, while the P50 of the second stimulus (S2) is related to the ability to inhibit other non-target stimuli in the presence of the first stimuli (Boutros and Belger, 1999; Bramon et al., 2004).

Abnormalities in sensory gating was first reported in patients with schizophrenia by Adler et al. (1982). Many studies (Jerger et al., 1992; Schwarzkopf et al., 1993; Smith et al., 1994; Griffith et al., 1995; Nagamoto et al., 1996; Jin et al., 1998; Boutros and Belger, 1999; Oranje et al., 1999; Light and Braff, 2000; Light et al., 2000; Olincy et al., 2000) have subsequently found that the P50 S2/S1 amplitude ratio of patients with schizophrenia is higher than that of healthy controls (HC), suggesting an impairment of sensory gating in schizophrenia. Many recent studies shifted their focus to the prodromal period, which is experienced by 80–90% schizophrenics with less intense symptoms but does not meet the diagnostic criteria for schizophrenia (Addington and Barbato, 2012). Individuals in this period are referred to as ultra-high risk individuals (UHR), also known as clinical risk individuals (Correll et al., 2010). Many studies have investigated whether the sensory gating deficits are present in UHR which may contribute to early diagnosis and intervention of schizophrenia. Indeed, some researches (Myles-Worsley et al., 2004; Brockhaus-Dumke et al., 2008) have found attenuated sensory gating deficits in UHR groups, while others (Hsieh et al., 2012; Ziermans et al., 2012; Van Tricht et al., 2015) found no significant differences in P50 parameters between UHR and other groups. Among them, Myles-Worsley et al. (2004) studied the auditory sensory gating in genetically high risk and UHR prodromal adolescents and found that auditory sensory gating was impaired in both groups. However, in genetically high risk group, abnormal P50 was only found in those with schizophrenia prodromal symptoms. Regarding FESZ, there are also inconsistent results on the P50 performance. Specifically, some studies (Yee et al., 1998; Brockhaus-Dumke et al., 2008) but not other studies (De Wilde et al., 2007; Hsieh et al., 2012; Van Tricht et al., 2015) have reported the sensory gating suppression in FESZ.

On the other hand, the underlying neurophysiological mechanisms of sensory gating and its disruption in schizophrenia are not completely clear. There are two main hypotheses to explain sensory gating deficits in schizophrenia. In the first hypothesis, gating disorders are believed to be attributed to a decline in the ability to adapt to repeated auditory stimuli (i.e., lager S2 amplitudes) (Adler et al., 1982; Clementz et al., 1998), while the second hypothesis states that gating impairments come from a decrease in sensory baseline (i.e., smaller S1 amplitudes) (Judd et al., 1992; Blumenfeld and Clementz, 2001; Johannesen et al., 2005; Arnfred, 2006; Brockhaus-Dumke et al., 2008).

Moreover, disturbances of the functional interactions between different brain regions have been found in schizophrenia (Dauvermann et al., 2014), which are considered to be the cause of cognitive impairment (Heinrichs and Zakzanis, 1998). The brain functional network can be constructed not only on data such as fMRI (Chen et al., 2019), but also based on EEG data (Liu et al., 2019). Several studies have calculated brain function connectivity at the scalp level and found abnormalities in schizophrenia, showing a reduced phase synchronization in the beta and gamma frequency ranges (Uhlhaas and Singer, 2010; Kikuchi et al., 2011; Kam et al., 2013; Yin et al., 2017), compared with HCs, but enhanced connectivity in the lower frequency bands (Kam et al., 2013). However, analysis of brain functional connectivity at the scalp level is limited due to volume conduction and reference electrode placement. Few previous studies (Andreou et al., 2015a, b) have reported functional brain networks based on source-level EEG data, almost invariably focused on other tasks, such as resting states or working memory. However, none of them studied the source-level brain functional network during the P50 sensory gating, which could provide reference for systematically observing information interactions between brain regions in the process of auditory gating. Critically, we currently have very little information about possible abnormalities before illness onset in UHR.

In order to explore the neurophysiological mechanisms of auditory gating, a few studies used source imaging to find the gating process generators. Early EEG-based studies (Reite et al., 1988; Huotilainen et al., 1998) consistently reported that the areas associated with auditory gating included the bilateral superior temporal gyrus, but suspected the existence of other sources. Later studies put forward a few hypothetical sources for the non-temporal part, including the frontal lobe (Weisser et al., 2001; Korzyukov et al., 2007), hippocampus, and thalamus (Huang et al., 2003). However, the neural mechanisms of sensory gating have not been fully clarified.

Identification of the neural networks involved in the auditory gating control deficits among first-episode schizophrenia patients (FESZ), UHR, and HC might help elucidate the pathophysiological mechanisms that induce the occurrence of auditory inhibition defects in schizophrenia and promote early diagnosis and intervention in UHR stage. Therefore, the main purpose of this study was to investigate the auditory sensory gating performance by evaluating the differences in brain functional network among FESZ, UHR, and HC. First, 128 channel scalp potentials during the P50 sensory gating were collected in the three groups. The scalp EEG data were transformed into cortical oscillation of brain electrical activity by eLORETA (Pascual-Marqui et al., 2011). The central source activity of 80 brain regions was used to represent the nodes of the brain functional network, which was built through mutual information. A pairwise comparison of the three groups in terms of functional networks was performed; then graph theory metrics, including average clustering coefficient (a global indicator) and characteristic path length (a local indicator) were evaluated. A decision tree model based on demographic characteristics, MATRICS Consensus Cognitive Battery (MCCB) parameters, event-related potential (ERP) profiles, and brain network connectivity indicators was then established.

## Materials and Methods

### Participants

The subjects of the study were outpatients of the Anding Hospital Affiliated to Capital Medical University. Subjects aged between 12 and 35 and with more than 6 years of education were selected. The Diagnostic and Statistical Manual of Mental Disorders, Fourth Edition (DSM- IV) (American Psychiatric Association, 1994)was used to diagnose SZ. FESZ patients were selected among those who had experienced their first psychotic episode. The UHR subjects met the criteria of the Structured Interview for Psychosis-Risk Syndrome, Criteria of Psychosis-risk Syndromes (SIPS-COPS) (Miller et al., 1999, 2002). Both FESZ and UHR had never used antipsychotic drug, or had taken antipsychotic medications for less than 1 month. The HCs were matched for age, gender, and education level, and had no blood relationships with the subjects in the other groups.

Criteria for exclusion were as follows: Diagnosis of delirium, dementia or other cognitive disorders; assessment by the Chinese version of the Wechsler Adult Intelligence showing clear intellectual impairment (IQ ≤ 70); researchers believing, based on the diagnostic procedure, that the subjects will commit suicide or violence; severe or unstable physical disease; electric twitch or magnetic stimulation received within 6 months. All SZ subjects with other psychiatric disorders were excluded from the FESZ group. For the other two groups, participants with a schizophrenia spectrum disorder, bipolar disorder, brain organic disorder, physical illness or psychoactive disorder were excluded. Individuals with impaired hearing were excluded from all the groups. The Ethics Committee of Beijing Anding Hospital reviewed and approved this study. All subjects provided informed consent prior to inclusion. In particular, we also obtained the written informed consent from the parents/legal guardians of any subject that is under the age of 16.

### Cognitive Assessment

The MATRICS Consensus Cognitive Battery (MCCB) (Shi et al., 2015) was assessed in a subsample of the subjects to assess cognitive function in the seven domains: Attention/vigilance, working memory, speed of processing, verbal learning, visual learning, reasoning and problem solving, and social cognition. The subsample, who participated in the assessment of cognitive performance by MCCB, including 25 FESZ patients (12 males and 13 females), 23 UHR individuals (11 males and 12 females), and 19 HC (11 males and 8 females), obtained from the total sample.

### EEG Recording

EEG recordings were obtained with Electrical Geodesics Inc. (Eugene, OR, United States) amplifiers with 128-channel Ag/AgCl electrode nets. Participants were placed in an electromagnetically shielded and noise-free room and seated comfortably. They were asked to stare at a black cross during P50 paradigm, which was displaying on a screen that was 55–65 cm from their eyes.

The sampling rate of EEG recording was 1000 Hz, and the collected data is bandpass filtered from 0.1 to 100. During the data acquisition, Cz was selected as the reference, and the resistance of each electrode was kept below 5 kΩ. Subjects were requested to listen through binaural headphones.

**FIGURE 1:**
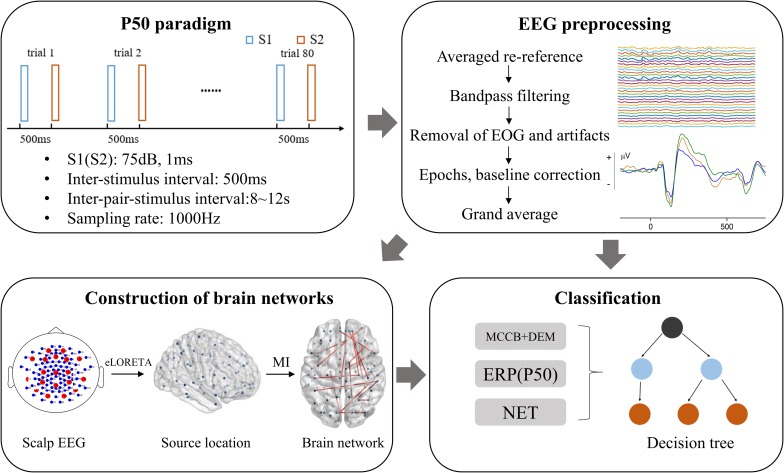
Flow chart of this study. MI, mutual information; NET, brain network properties. DEM, demographic data; MCCB, MATRICS Consensus Cognitive Battery; EOG, electro-oculogram; eLORETA, exact low-resolution electromagnetic tomography.

P50 is a voluntary, pre-attentional ERP induced by paired-click paradigm with no task required. The formal P50-induced auditory experiment consists of two same sessions, each containing 40 pairs of identical clicks. The stimulus interval is 500 ms and the interval between pair-stimulus (trials) is random 8–12 s. The first stimulus in each pair of stimuli is referred to as stimulus 1 (S1), and the second is correspondingly referred to as stimulus 2 (S2). S1 and S2 have a duration of 1ms and a sound intensity of 75 db. The flow chart of this study is presented in Figure 1, which contains a schematic of the P50 paradigm.

### Data Analysis

#### Preprocessing

A series of offline preprocessing steps including average re-referencing, 1–40 Hz bandpass filtering, removal of electro-oculogram (EOG) and movement artifacts, data segmentation and baseline correction, were performed with MATLAB^1^ and the open-source toolbox EEGLAB^2^. EOG was removed using independent component analysis (ICA) (Makeig et al., 1997; Vigário, 1997). Each ERP trial was manually checked, which contains drift and movement artifacts was rejected.

Epochs were extracted from 200 ms before the onset of stimulus 1 onset to 1,000 ms following stimulus 1 presentation, with the first 200 ms used for baseline correction. EEG data were averaged across all trials for each participant. The P50 was identified as the maximum positive peak between 30 and 90 ms after stimulus onset. Four individuals were excluded from the analysis, since their P50 amplitudes in response to S1 did not exceed 0.5 mV. The segments from 30 to 90 ms after stimulus 1 (S1) and stimulus 2 (S2) onset, which were used to conduct the following brain functional network analysis, were taken to represent the S1 and S2 P50 components, respectively. The S1 wave was chosen to represent the baseline. The pointwise subtraction of the S2 wave, introduced in a previous study (Korzyukov et al., 2007), was used to represent the gating response for further endogenous reconstruction.

#### Endogenous Reconstruction

A recent study (Jatoi et al., 2014) showed that eLORETA has better traceability and higher ability to inhibit low-significance sources, compared with sLORETA (Pascual-Marqui, 2002), when analyzing high-time resolution stimulation-induced ERP signals. Another advantage of eLORETA is that, even in the case of structural noise, it has no location deviation (Pascual-Marqui, 2007).

In this paper, the LORETA software^3^ was used to trace the scalp EEG 128-channel signal to cortical signal of 6329 voxels. However, due to the low spatial resolution of eLORETA, neighboring neuron sources are highly correlated. Thus, the central voxels of the 80 brain regions (40 for each hemisphere, see Supplementary Table 1 for the coordinates of each position), referred to the Automated Anatomic Labeling atlas, were chosen to represent the activity of their brain regions (Andreou et al., 2015a, b).

Hence, there were $C_{80}^{2}=3160$ pairs of nodes to be assessed by correlation analysis, sufficient to calculate reliable brain connectivity under the limitations of the spatial resolution of the EEG. An 80 × 80 undirected weighted correlation matrix was thus constructed. To test the differences between pair of groups, $3\,C_{80}^{2}=9480$ combinations were computed for every connectivity by custom procedures written in MATLAB.

#### Mutual Information

Mutual information (Xu et al., 1997), based on the theory of entropy, is used to quantify the correlation between two signals. Mutual information can effectively measure the interaction and dynamic characteristics of information transmission between two signals. Moreover, in contrast with coherence, phase synchronization, and so on, mutual information can be applied to signals in specific frequency bands, as well as signals in the full frequency band. In addition, due to the simplicity of the calculations and the low data length requirements, mutual information more often used to quantify the relationship between signals. For these reasons, mutual information has been applied in the study of a number of brain diseases, such as Alzheimer’s disease (Jeong et al., 2001; Liu et al., 2014), schizophrenia (Huang et al., 2013; Yin et al., 2017) and epilepsy (Jeong et al., 2014), with impressive results.

The value of the mutual information describes the transfer of information between the reconstructed cortical time series and further reveals the closeness of the connections between different brain regions. Mutual information can be applied to signal analysis in the full frequency band of EEG, as well as in a specific frequency band, to examine the information contained in both the amplitude and the phase of the signal. The MI of two source signals is defined as:

MI⁢(S,Q)=H⁢(S)+H⁢(Q)-H⁢(S,Q)

Where S and Q are two EEG time series, H(S) and H(Q) represent their information entropies, and H(S, Q) is their joint information entropy.

The MI is standardized to obtain the normal mutual information (NMI):

NMI⁢(S,Q)=H⁢(S)+H⁢(Q)-H⁢(S,Q)H⁢(S)⁢H*⁢(Q)

Based on the above definition, we can calculate the mutual information between any two sources. In this way, when the signals of the two brain regions transmit information without interference and loss, their mutual information is 1; when there is no information transmission between the signals of the two brain sources, the value of their mutual information is 0; other situations are somewhere between 0 and 1.

#### Network Properties

After constructing the undirected weighted network, network indexes were calculated using graph theory. In order to measure the characteristics of the network from different perspectives, we selected indicators describing both global (i.e., characteristic path length and global efficiency) and local (i.e., average clustering coefficient) features. These parameters are described below.

##### The characteristic path length

The characteristic path length describes the shortest path for information flow between two nodes in the network, through which information can be transmitted fastest. The characteristic path length of a network is the average of the characteristic path length between all pairs of nodes in the network, thus defined as:

$$L=\frac{1}{n\,\left(n-1\right)}\,\sum_{i,j\in V,i\neq j}l_{\mathrm{ij}}$$

where *n* is the total number of nodes, and *l*_*ij*_ is the characteristic path length between nodes *i* and *j*. In this study, *n* is equal to 80, corresponding to 80 brain regions that are the nodes of the network.

##### The average clustering coefficient

The clustering coefficient measures the degree of grouping of the network and is an important parameter for network characterization. The ratio of the number of edges actually connected between neighbors of a node *i* to the maximum number of possible connected edges is defined as the clustering coefficient of node *i*. The average clustering coefficient represents the functional segregation ability of the brain. It is defined as:

$$C=\frac{1}{n}\,\sum_{i\in V}C_{i}=\frac{1}{n}\,\sum_{i\in V}\frac{E_{i}}{k_{i}\,\left(k_{i}-1\right)}$$

where *C*_*i*_ represents the clustering coefficient of node i (*C*_*i*_ = 0 for *k*_*i*_ < 2). *k*_*i*_ represents the degree of node *i*, and

$$E_{i}=\sum_{j,h\in V,j\neq i\neq h}a_{\mathrm{ij}}\,a_{\mathrm{hi}}\,a_{\mathrm{hj}}$$

where *a*_*ij*_, *a*_*hi*_ and *a*_*hj*_ are the relevant elements of the adjacency matrix.

##### Global efficiency

The global efficiency is a parameter that measures the ability to integrate information among regions of the brain, and is defined as:

$$E_{\mathrm{global}}=\frac{1}{n\,\left(n-1\right)}\,\sum_{i\neq j\in V}l_{\mathrm{ij}}$$

where *l*_*ij*_ is the shortest path length between nodes *i* and *j*, *V* is a set of nodes, and *n* is the total number of nodes in set *V*.

#### Classification

The decision tree (Quinlan, 2014) is one of most classic classifiers in machine learning which is a non-parametric supervised learning method used for classification and regression. The decision tree, including branches and nodes, uses the structure of the tree to classify the data, and builds the lower nodes and branches according to the subset of each branch to generate a decision tree model.

Subjects participating in the classification were the same as those in the MCCB test, a total of 67, including 25 FESZ, 23 UHR, and 19 HC. A total of 24 features were extracted from the previous analysis as the input features to be used to construct a decision tree. These features were divided into 4 classes: demographic data (DEM), brain network parameters (NET), MCCB indexes, and P50 ERP performance (ERP). Among them, demographic data included age and years of schooling. The clustering coefficients, feature path lengths, and global efficiencies of S1, S2, and S1-S2 were the features chosen among the brain network parameters. As mentioned above, there are seven indexes in MCCB, which were all included among the features. The P50 ERP features consisted of the amplitude of S1, the amplitude of S2, the difference S1-S2 and the ratio S2/S1.

The classifications for differentiating the three groups were carried out three times in total: In the first time classification only demographic characteristics and MCCB parameters were used; in the second time classification ERP features combined with MCCB parameters were used to training the classifier. The brain network parameters, i.e., the main parameters obtained in this study, were added with ERP features and MCCB parameters together in the third time classification. The classification results of three times were studied separately, including the classification accuracy, the correlation between the parameters, and the importance weights of the various features.

We used post-pruning, analyzing performance on cross-validation sets and pruning child nodes, to effectively prevent over-fitting. In each classification, we randomly selected 20% of the samples in each group (FESZ, UHR and HC) for testing, and the remaining 80% of the samples were used for training the classifier model. Specifically, 5 samples were randomly selected from 25 FESZs, 5 randomly selected from 23 UHRs, and 4 randomly selected from 19 HCs, all of the 14 samples form a test set; and the remaining 53 samples form a training set. For the classification with different combinations of input features (DEM, MCCB; DEM, MCCB and ERP; DEM, MCCB, ERP and NET), we used the randomly selected training set and test set using the method described above, and performed 101 times independently to evaluate the performance of the method by accuracy. The classification results were represented by the median of the 101 accuracy values for each classifier, and were presented in the form of a confusion matrix.

A stratified fivefold cross-validation scheme was used to further reduce over-fitting and evaluate the classification performance of the model. In this program, the original data set was divided into fivefold of the same size, one for testing and the other four for training. This process was repeated five times, that is, the fivefold were traversed as a test set. And five results were averaged to produce a single performance metric estimate. To assess consistency, the same architectural setup and training from scratch were used for each iteration of five cross-validations. In our study, the original data set consist 67 samples, including 25 FESZ, 23 UHR, and 19 HC. Thus, we divided the original sample into fivefold of 13, 13, 13, 14, and 14.

#### Statistical Analysis

Analysis of variance (ANOVA) and chi squared tests were used to compare pairs of groups in terms of demographic characteristics. The non-parametric Kruskal–Wallis test was used to assess differences among groups (FESZ, UHR and HC) in terms of P50 amplitudes, amplitude difference S1-S2, amplitude ratio S2/S1, and the parameters of the brain network. *Post hoc* tests were performed with the Bonferroni method. Statistical analysis was performed using SPSS software (version 22). The differences among three classifiers using the same set of features (DEM, MCCB; DEM, MCCB and ERP; DEM, MCCB, ERP, and NET) were measured by independent sample *T*-test.

## Results

### Demographic and Cognitive Characteristic

The demographic and cognitive characteristics of our sample are summarized in Table 1. There were no between-group differences in age (*F* = 2.947, *p* = 0.057), gender (χ^2^ = 4.879, *p* = 0.087), and education (*F* = 2.626, *p* = 0.077). In addition, we also performed a demographic analysis using the same method in the subsample with MCCB evaluation. Also in this subset of the subjects there were no significant difference among the groups in terms of age (*F* = 2.453, *p* = 0.094), gender (χ^2^ = 0.542, *p* = 0.762), and education (*F* = 0.030, *p* = 0.970; these data were not included in Table 1).

**TABLE 1:** Demographic, cognitive and performance characteristics of the study sample.^a^

**Characteristic**	**FESZ①**		***UHR*②**		**HC③**		**Comparison**		***Post hoc* p-values**		
	***N***	**%**	***N***	**%**	***N***	**%**	**χ^2^/F**	**p**	**① vs. ②**	**① vs. ③**	**② vs. ③**

Gender(female/male)	20/15	57.1/42.9	15/15	50/50	13/27	32.5/67.5	4.879	0.087			

	**Mean**	***SD***	**Mean**	***SD***	**Mean**	***SD***					
Age(years)	25.09	6.44	22.67	5.38	25.45	2.90	2.947	0.057	0.167	1.000	0.072
Education(years)	12.74	3.08	13.37	3.12	14.38	3.14	2.626	0.077	1.000	0.077	0.550
**Cognition(subsample^b^)**											
Speed of processing	33.28	9.22	36.96	7.21	41.68	6.38	6.228	**0.003**	0.326	**0.002**	0.167
Attention/vigilance	31.91	10.63	39.53	9.04	47.79	8.80	14.164	**0.000**	**0.049**	**0.000**	**0.039**
Verbal learning	39.12	9.83	42.74	10.08	46.58	9.34	3.151	**0.050**	0.615	**0.044**	0.630
Working memory	37.24	10.01	40.70	9.35	44.84	8.07	3.631	**0.032**	0.605	**0.027**	0.462
Visual learning	40.96	13.77	44.04	10.22	43.89	10.07	0.517	0.599	1.000	1.000	1.000
Reasoning/problem solving	34.32	11.87	37.74	9.21	40.37	10.16	1.823	0.170	0.797	0.191	1.000
Social cognition	30.80	11.79	38.55	7.90	37.00	11.06	3.626	**0.033**	**0.040**	0.188	1.000
Overall composite	35.22	7.08	39.83	6.02	42.88	6.40	6.912	**0.002**	0.089	**0.002**	0.527
**P50 measure**											
S1 amplitude(μV)	1.24	0.58	1.13	0.73	1.30	0.90	1.212	0.546	0.533	0.731	0.924
S2 amplitude(μV)	0.99	0.61	0.67	0.43	0.74	0.56	7.081	**0.029**	**0.019**	**0.025**	0.772
S1-S2(μV)	0.25	0.54	0.41	0.79	0.55	0.82	1.397	0.497	0.824	0.464	0.860
S2/S1	0.87	0.50	0.78	0.68	0.65	0.43	5.157	0.076	0.282	0.068	0.836

*^*a*^Number and percentage of subjects are shown for gender, analyzed by chi squared test. Group means and standard deviations (SD) are reported for age, years of education and the MATRICS Consensus Cognitive Battery, analyzed by ANOVA. ^*b*^The subsample, who participated in the assessment of cognitive performance by MCCB, including 25 FESZ patients (12 males and 13 females), 23 UHR individuals (11 males and 12 females), and 19 HC (11 males and 8 females), obtained from the total sample. *P*-values of less than 0.05 in the table are marked with bold.*

The MCCB performance indexes are summarized in Table 1. Their statistical analysis shows that visual learning (*F* = 0.517, *p* = 0.599) and reasoning/problem solving (*F* = 0.170, *p* = 0.797) did not differ significantly among the three groups. However, there were significant differences among groups in the other domains, including speed of processing, attention/vigilance, verbal learning, working memory, social cognition, and overall composite index. In particular, *post hoc* testing showed a significant difference in attention/vigilance between all three group pairs. On the contrary, speed of processing, verbal learning, working memory, and the overall composite index were revealed by *post hoc* tests to be significantly poorer in FESZ compared with HC, whereas UHR did not significantly differ from either FESZ or HC. FESZ showed significantly worse social cognition than UHR, but there was no significant difference comparing HC with FESZ and HC.

### ERP Component

The P50 is induced by the classic paradigm to assess auditory sensory gating, usually distributed around brain parietal lobe. Thus, the electrodes 6, 7, and 106 around the Cz site (the distribution of all electrodes is shown in the Supplementary Figure 1) were chosen to represent the ERP data after averaging all valid trials.

**FIGURE 2:**
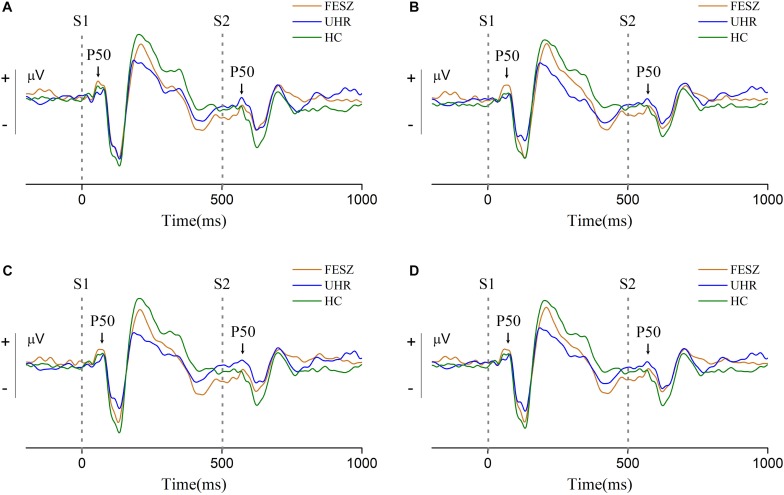
Grand-average event-related potential waveforms at sites 6, 7, and 106 around Cz (site 7 is located at the upper left of the adjacent Cz, site 106 at the upper right, and site 6 is located above the middle of sites 7 and 106. The distribution of all electrodes is shown in the Supplementary Figure 1). **(A–C)** shows the grand-average event-related potential waveforms of sites 6, 7, and 106, respectively, and **(D)** shows their sum. Two gray dotted lines in each subgraph represent stimulus 1 (S1) and stimulus 2 (S2) respectively. The S1 and S2 component of P50 are indicated with arrowheads. In each subgraph, the orange line represents FESZ, the blue line represents UHR, and the green line represents HC.

Grand-average event-related potential waveforms for each group are shown in Figure 2. The S1 and S2 subcomponent of P50 are indicated with arrowheads. Following the P50 component, the obvious auditory components N100 and P200 can be observed in all three groups, indicating that the primary auditory process was successfully induced. We observed the ERP waveforms on three different sites with similar trends, indicating that the area associated with the P50 component was successfully activated. For the P50 induced by stimulus 1, shown in Figure 2, the amplitude in the FESZ was greater than in the other two groups, which were very similar to each other. The P50 induced by stimulus 2 was similar to that of stimulus 1, and can be clearly seen to be significantly larger in FESZ than in the other two groups, which are similar to each other and not easily distinguished.

The non-parametric Kruskal–Wallis test was performed to assess the differences in performance of the P50 component among the three groups. The P50 amplitude of stimulus 1 did not differ among groups [χ^2^ of Kruskal–Wallis test = 1.212, degree of freedom (df) = 2, *p* = 0.546], while the P50 amplitude of stimulus 2 showed significant differences among the groups (χ^2^*ofKruskal*−*Wallistest* = 7.081, df = 2, *p* = 0.029). Difference and ratio between S1 and S2 were also calculated and analyzed with the Kruskal–Wallis test. However, there were no statistical differences among groups in S1–S2 (χ^2^ of Kruskal–Wallis test = 1.397, df = 2, *p* = 0.497) or S2/S1 (χ^2^ of Kruskal–Wallis test = 5.157, df = 2, *p* = 0.076).

### Connectivity Differences Between Groups

**FIGURE 3:**
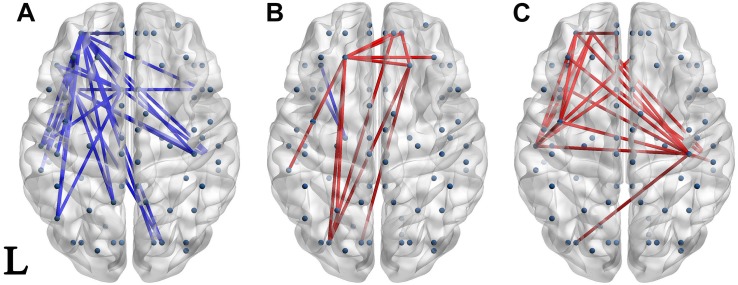
Network of significantly different connectivities in the three groups from the S1 ERP waveform. Significantly different connectivities in the three groups are shown: **(A)** FESZ vs. UHR; **(B)** FESZ vs. HC; **(C)** UHR vs. HC. A blue (red) line represents significantly lower (higher) connectivity in the first group compared with the second. For example, the blue lines in **(A)** indicates lower connectivities of FESZ compared with UHR. The connectivities was displayed by BrainNet Viewer (http://www.nitrc.org/projects/bnv/).

**FIGURE 4:**
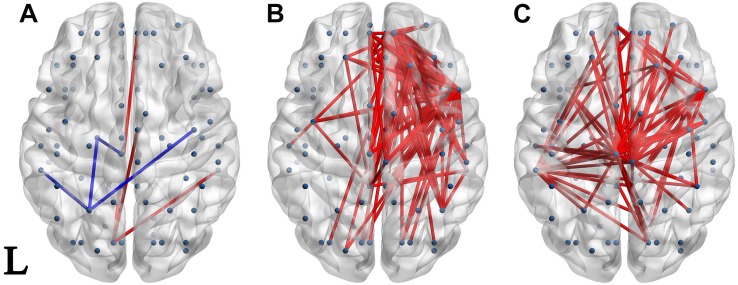
Network of significantly different connectivities in the three groups from the S1-S2 ERP waveform. Significantly different connectivities in the three groups are shown: **(A)** FESZ vs. UHR; **(B)** FESZ vs. HC; **(C)** UHR vs. HC. A blue (red) line represents significantly lower (higher) connectivity in the first group compared with the second. For example, the blue lines in **(A)** indicates lower connectivities of FESZ compared with UHR. The connectivities was displayed by BrainNet Viewer (http://www.nitrc.org/projects/bnv/).

Significantly different connectivities between every pair of groups were observed by permutation test (*p* < 0.05). Those based on the S1 and S1-S2 waveforms are displayed in Figures 3, 4 respectively.

The networks of significantly different connectivities among the three groups, obtained from the S1 ERP waveform, are shown in Figure 3. There were relatively few significantly different connections derived from the S1 ERP waveform in the three comparisons. The key nodes involved in the connection between FESZ and UHR were in the left hemisphere, and mainly in the temporal pole, superior temporal gyrus and middle orbitofrontal cortex. Moreover, all connectivities were weaker in FESZ than in UHR. The key nodes involved in the different connections between UHR and HC were mainly in the right and left postcentral gyrus, left superior temporal gyrus and middle orbitofrontal cortex. The UHR connectivities were higher compared with HC.

There were few significantly different connections from the S1-S2 ERP waveform between FESZ and UHR, and more in the other two group pairs. The key nodes involved in the different connectivities between FESZ and HC were in the right hemisphere, mainly including the superior frontal gyrus, orbital part, insula, inferior frontal operculum, and media orbitofrontal cortex. All connectivities were enhanced in FESZ compared with HC. The key nodes involved in the different connectivities between UHC and HC are in the paracentral lobule, left supramarginal gyrus, and right insula, and the connections were stronger in UHR than in HC. The degree of the significantly different connected regions nodes in the FESZ vs. HC and UHR vs. HC based on S1-S2 is shown in Table 2.

**TABLE 2:** The degree of the significantly different connected nodes in the FESZ vs. HC and UHR vs. HC based on S1-S2.

**FESZ vs. HC**			**UHR vs. HC**		
**Region**	**Hemisphere**	**Degree**	**Region**	**Hemisphere**	**Degree**
Superior frontal gyrus, orbital part	Right	34	Paracentral lobule	Right	21
Insula	Right	22	Paracentral lobule	Left	21
Inferior frontal operculum	Right	19	Middle temporal gyrus	Left	18
Media orbitofrontal cortex	Right	15	Insula	Right	12
Superior frontal gyrus	Right	10	Inferior frontal operculum	Right	9
Posterior cingulate cortex	Left	10	Media orbitofrontal cortex	Right	6
Angular gyrus	Right	8	Temporal pole, middle temporal gyrus	Left	6
Superior frontal gyrus, medial part	Left	8	Precentral gyrus	Right	5
Middle temporal gyrus	Right	8	Superior frontal gyrus	Right	5
Paracentral lobule	Right	7	Posterior cingulate cortex	Right	5
Posterior cingulate cortex	Right	5	Media orbitofrontal cortex	Left	5
Paracentral lobule	Left	4	Inferior parietal lobule	Right	4
Precentral gyrus	Left	4	Rolandic operculum	Right	4
Superior frontal gyrus	Left	4	Superior frontal gyrus	Left	3
Rolandic operculum	Right	4	Cuneus	Right	3
Gyrus rectus	Left	4	Lingual gyrus	Right	3
Middle frontal gyrus	Right	3	Superior parietal lobule	Left	3
Cuneus	Right	3	Middle frontal gyrus	Right	2
Superior frontal gyrus, medial part	Right	3	Calcarine sulcus	Right	2
Anterior cingulate cortex	Right	3	Superior temporal gyrus	Left	2
Middle cingulate cortex	Right	2	Superior frontal gyrus, orbital part	Right	2
Supramarginal gyrus	Right	2	Parahippocampal gyrus	Left	2
Superior occipital gyrus	Left	2	Inferior parietal lobule	Left	1
Calcarine sulcus	Left	2	Angular gyrus	Right	1
Middle orbitofrontal cortex	Right	2	Superior frontal gyrus, medial part	Left	1
Olfactory gyrus	Right	2	Supramarginal gyrus	Left	1
Supplementary motor area	Left	1	Middle orbitofrontal cortex	Right	1
Superior occipital gyrus	Right	1	Superior frontal gyrus, orbital part	Left	1
Cuneus	Left	1			
Middle occipital gyrus	Left	1			
Superior temporal gyrus	Left	1			
Inferior frontal gyrus, orbital part	Right	1			
Inferior occipital gyrus	Right	1			
Temporal pole, superior temporal gyrus	Right	1			
Parahippocampal gyrus	Right	1			

*Regions are ordered by decreasing degree.*

The brain regions at both ends of the significantly different connections based on S1-S2 in the UHR vs. HC comparison were identified, and the top 35 connections are shown in Table 3, ordered by *P*-value.

**TABLE 3:** Regions involved in the top 35 significantly different functional connectivities based on S1-S2 ERP waveform in the UHR vs. HC comparison.

**Region 1**		**Region 2**		***P***
Right	Paracentral lobule	Right	Middle temporal gyrus	0.0007
Right	Paracentral lobule	Left	Inferior frontal operculum	0.0008
Right	Superior frontal gyrus	Right	Middle temporal gyrus	0.0009
Right	Paracentral lobule	Right	Temporal pole, middle temporal gyrus	0.0009
Left	Paracentral lobule	Right	Temporal pole, middle temporal gyrus	0.0009
Right	Paracentral lobule	Left	Insula	0.0013
Left	Precentral gyrus	Right	Middle temporal gyrus	0.0014
Left	Paracentral lobule	Right	Middle temporal gyrus	0.0015
Left	Paracentral lobule	Left	Superior frontal gyrus	0.0016
Right	Paracentral lobule	Left	Rolandic operculum	0.0020
Left	Paracentral lobule	Left	Inferior frontal operculum	0.0020
Right	Paracentral lobule	Right	Media orbitofrontal cortex	0.0021
Left	Insula	Right	Middle temporal gyrus	0.0024
Right	Paracentral lobule	Left	Inferior parietal lobule	0.0024
Left	Insula	Left	Posterior cingulate cortex	0.0026
Left	Paracentral lobule	Left	Superior frontal gyrus, orbital part	0.0027
Left	Paracentral lobule	Left	Inferior parietal lobule	0.0027
Left	Insula	Right	Media orbitofrontal cortex	0.0030
Left	Middle temporal gyrus	Right	Posterior cingulate cortex	0.0030
Left	Inferior frontal operculum	Right	Media orbitofrontal cortex	0.0030
Right	Paracentral lobule	Left	Superior frontal gyrus	0.0030
Left	Inferior frontal operculum	Left	Media orbitofrontal cortex	0.0031
Right	Paracentral lobule	Left	Precentral gyrus	0.0033
Left	Paracentral lobule	Left	Rolandic operculum	0.0033
Right	Middle temporal gyrus	Left	Lingual gyrus	0.0035
Right	Middle temporal gyrus	Right	Media orbitofrontal cortex	0.0036
Left	Rolandic operculum	Left	Insula	0.0039
Left	Paracentral lobule	Right	Media orbitofrontal cortex	0.0039
Left	Paracentral lobule	Left	Insula	0.0039
Left	Paracentral lobule	Left	Angular gyrus	0.0039
Left	Paracentral lobule	Right	Paracentral lobule	0.0040
Left	Paracentral lobule	Right	Superior frontal gyrus, medial part	0.0041
Right	Superior parietal lobule	Right	Middle temporal gyrus	0.0044
Right	Paracentral lobule	Left	Middle frontal gyrus	0.0045
Left	Paracentral lobule	Left	Precentral gyrus	0.0046

### Classification

**FIGURE 5:**
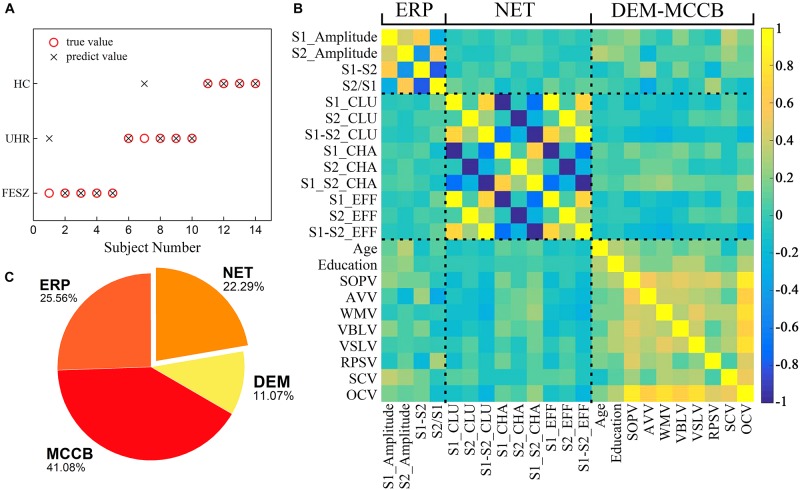
A series of results from one of the classification with ERP, MCCB, DEM and NET as input features including **(A)** Predictions of the third stage decision tree classifier including all 24 features as inputs. The abscissa represents 14 subjects for which the group was predicted. Black crosses represent predicted values and red circles show the true values. **(B)** Pearson correlation matrix between the features used in the final classifier. A total of 24 features from left to right of the *x*-axis are divided into 4 classes: P50 ERP behavior (including S1_Amplitude, S2_Amplitude, S1-S2 and S2/S1), brain network parameters (S1_CLU, S2_CLU, S1_S2_CLU, S1_CHA, S2_CHA, S1_S2_CHA, S1_EFF, S2_EFF and S1_S2_EFF), demographic data (gender, age and education), and MCCB (SOPV, AVV, WMV, VBLV, VSLV, RPSV, SCV and OCV). CLU: clustering coefficient; CHA: characteristic path length; EFF: efficiency. SOPV: speed of processing; AVV: attention/vigilance; WMV1: working memory; VBLV: verbal learning; VSLV: visual learning; RPSV: reasoning and problem solving; SCV: social cognition; OCV: Overall composite. The rightmost color bar from deep blue to light yellow represents a gradient from −1 to 1 in correlation. **(C)** A pie chart revealing the importance of the four types features. The features represented by each color and its proportions are shown in the figure.

We selected the specific features to be used in three decision tree classifications, divided into four classes, namely demographic data (DEM), brain network parameters (NET), MCCB and P50 ERP performance (ERP). The accuracy of the three different classifiers is summarized in Table 4. The first stage classification only used DEM and MCCB to classify the three groups: FESZ, UHR and HC. The mean accuracy of this first classifier was merely 53.90%. The second classification added the ERP performance, and the mean accuracy increased to 64.29%. In the final stage, brain network parameters based on graph theory were added, and the mean accuracy of 79.22% was achieved. The accuracy of the combination of features DEM, MCCB and ERP as the input of the classifier is significantly higher than that obtained without the features ERP (*t* = 2.589, *p* = 0.018). Similarly, the accuracy of the combination of features DEM, MCCB, ERP and NET as the input of the classifier is significantly higher than that obtained without the features NET (*t* = 3.516, *p* = 0.002). The detailed results obtained from the classification with DEM, MCCB, ERP and NET as input are shown in Figure 5. The results of classification using the fivefold cross-validation with three features combination as input are shown in Table 5. In this study, the 24 features thus provided great accuracy 92.86% for the classification of first-episode schizophrenia, ultra-high risk individuals and HC.

**TABLE 4:** Accuracy of 101 times classification with three different combinations as input features of decision tree, and confusion matrix of the classification represented by the median of the 101 accuracy values by subject class (FESZ, UHE and HC).

	**Accuracy**	**FESZ**	**UHR**	**HC**	**Total**	
					**P/T**	**%**
DEM, MCCB	53.90 ± 9.78	3/5	2/5	3/4	8/14	57.14
DEM, MCCB, ERP	64.29 ± 9.03^∗^	3/5	4/5	2/4	9/14	64.29
DEM, MCCB, ERP, NET	79.22 ± 10.81^∗^	4/5	4/5	4/4	12/14	85.71

*^∗^Indicates a significant difference compared to the previous group, *p* < 0.05. The denominator (T) represents the number of samples in the test set, and the numerator (P) represents the number of samples that are predicted correctly. DEM, demographic data; NET, brain network parameters; MCCB, MCCB indexes; ERP, P50 performance.*

**TABLE 5:** The results of classification using the fivefold cross-validation with three features combination as input.

	**DEM, MCCB (%)**	**DEM, MCCB, ERP (%)**	**DEM, MCCB, ERP, NET (%)**
1	42.86	69.23	69.23
2	50	64.29	78.57
3	38.46	76.92	84.62
4	53.85	57.14	64.29
5	61.54	61.54	92.31
Mean	49.34	65.82	77.80

The classification results in Table 4 represented by the median of the 101 accuracy values for each combination of features as input were also displayed. With DEM, MCCB, ERP and NET as input features, of the 14 predicted subjects, only two were assigned to the wrong category, and all HCs were correctly predicted, proving the reliability of the classifier when using all four kinds of features.

The average accuracy by fivefold cross-validation with three combinations of features as input of the decision tree shows the improvement of performance by the addition of electrophysiological features.

The Pearson correlation matrix between the features used in the classification is shown in Figure 5B. Highly correlated features are clustered near the diagonal of the correlation matrix, and the clusters from the lower left to the upper right represent P50 ERP performance, brain network parameters, demographic data and MCCB, indicating that the aggregation effects appeared among related attributes.

In addition to the above results on classification, feature importance was calculated according to four major characteristics shown in Figure 5C. The MCCB parameters accounted for a relative importance of 41.08%, the ERP component contributed 25.56%, demographic characteristics 11.07% and the functional brain network 22.29%. Therefore, it can be concluded that the characteristics of brain functional network based on graph theory play a significant role in the classifier.

## Discussion

This study investigated the differences in brain functional connectivity during the P50 sensory gating between unmedicated UHR, and drug-free FESZ and HC, separately. Our results showed that there were only a few significantly different connectivities among UHR, FESZ, and HC at baseline (i.e., S1 period). In contrast, in the gating response stage (i.e., the S1-S2 period), there were a large number of significantly different connectivities between UHR and HC. However, there were almost no significantly different connectivities between FESZ and UHR. In addition, we extracted the brain network characteristic parameters (including clustering coefficient, characteristic path length, and efficiency) based on graph theory from networks of the three groups, and the addition of these parameters improved the accuracy of the classifier. Our findings suggest that significantly different P50 functional network connectivities could improve the early identification and diagnosis of schizophrenia and provide insight into the mechanisms of P50 suppression in schizophrenia.

### ERP Component

The amplitude of the S1 component of P50 ERP represents the normal auditory response, i.e., the auditory baseline of every individual. The S2 amplitude indicates the degree of inhibition of the response to the second stimulus in the presence of the first stimulus. Moreover, the ratio S1/S2 is a relative value representing the auditory gating inhibition after the first stimulus (S1). Likewise, the difference S1–S2 represents the extent of suppression after removing the baseline. The S2 amplitude was significantly different among the three groups, yet S1/S2 and S1–S2 were not, indicating differences in auditory gating among the three groups. The lack of significant differences after taking the ratio or subtracting S1 from S2 might be due the relatively small amplitude range. However, this result also supports the assumption that the difference in auditory gating mainly comes from the response to the S2 stimulation, consistent with many previous studies (Adler et al., 1982; Clementz et al., 1998).

In addition to the above reasons, another cause of the poor performance of the ERP component is related to the collection device. In this study, Electrical Geodesics Inc. amplifiers, based on the Cz site as the reference electrode, were used to collect EEG signals, causing the EEG signal collected by the electrodes near Cz to be weaker than that of electrodes placed far from Cz. Although we added the average reference electrode as a re-reference in the offline pre-processing, the effect of the Cz reference electrode was not completely eliminated. Unfortunately, the P50 component performs best near Cz, and the electrodes chosen to measure P50 performance in this study were around Cz sites. As a result, the S1 and S2 amplitudes in this study were much smaller than those in other studies.

In addition, an study published this year (Hsieh et al., 2019) also reported P50 performance in FESZ, UHR and HC. The result showed that there was no significant difference among FESZ, UHR and HC in P50 performance (including S1 amplitude, S2 amplitude, P50 ratio and P50 difference). A review (Lepock et al., 2018) of ERP in the UHR state summarized the cognitive ERP studies and also pointed out that P50 performance appear to be less stable in UHR and FESZ. And in between-group and within-group comparisons, the trend is consistent with those observed in previous studies (Hamilton et al., 2018), so that our results can be considered reliable, and demonstrate that the method proposed in this paper can effectively measure the features we want to observe also in the case of weaker signals.

### Connectivity Differences Between Groups

Although the differences in ERP are not obvious, a clearer result can be observed from the brain functional networks. At baseline (i.e., the S1 period), the connections of the node representing the left superior temporal gyrus were significantly enhanced in UHR, indicating that the left supratemporal regions in UHR were more active than those in FESZ and HC. A large number of studies (Chatrian et al., 1960; Cohen, 1982; Lee et al., 1984; Liegeois-Chauvel et al., 1994) have proved that the superior temporal gyrus directly produces much of the P50 component. A previous study (Huang et al., 2003) had shown that the bilateral supraorbital gyrus P50 generators in HC accounted for 97% of the signal variance during 30–100 ms after the first click, whereas it was significantly lower (86%) in schizophrenia patients, which indicated that P50 generators had been damaged in schizophrenia. In this regard, compared with FESZ and HC, the enhancement of the superior temporal gyrus connection in UHR could be explained by that the generator damage had occurred in the UHR stage and the response degree was higher than that of FESZ.

In our study, there was little difference in the gating response stage (i.e., the S1–S2 period) between the UHR and FESZ, and both groups showed higher connectivities than the HC. In terms of ERP performance, there were no significant differences in the gating-related indicators between FESZ and UHR (S1–S2: *p* = 0.352, S1/S2: *p* = 0.548). This result may be because UHR are close to FESZ in brain function during the gating period. In addition, both FESZ and UHR had more significantly different connectivities with HC during the gating period; however, the regions involved were inconsistent: the difference between FESZ and HC mainly appeared in the right superior frontal gyrus and right insula; the difference between UHR and HC mainly appeared in the paracentral lobule and middle temporal gyrus. Many studies (Weisser et al., 2001; Oranje et al., 2006; Korzyukov et al., 2007; Jensen et al., 2008) have identified frontal sources involving P50 suppression in schizophrenia. Another study (Bak et al., 2011) reported that the insula was involved in the inhibitory processes of P50 sensory gating. Which are consistent with the regions involved different connectivities during gating period in our study between FESZ and HC. In summary, we speculate that, compared with HC, impaired brain regions in FESZ and UHR are different: the superior frontal gyrus and insula in FESZ, and the paracentral lobule and middle temporal gyrus in UHR.

Beyond brain functional deficits in schizophrenia, a study on brain structural images of schizophrenia (van den Heuvel et al., 2010) also showed impaired connectivity between the frontal and temporal brain regions. In our study, both the UHR and the FESZ showed enhanced connections between brain regions compared with the HC, and thus the connections were stronger than in healthy individuals.

A recent study (Van Tricht et al., 2015) supports the hypothesis that UHR are impaired mostly at baseline than during the sensory gating period. However, in our study, the result is exactly the opposite whether from the perspective of ERP performance or brain functional network. In other words, our findings support the hypothesis that impairment in P50 sensory gating in UHR occurs mostly in sensory gating period rather than at baseline.

In summary, we speculate that the superior temporal gyrus is related to the baseline and participates in the primary auditory information processing; the insula, paracentral lobule, and middle temporal gyrus are related to the gating and participate in a more advanced phase of auditory information processing. The results of this paper suggest that UHR had suffered varying degrees of damage both in the primary and, more significantly, in the advanced auditory information processing stages.

### Classification

Besides being directly displayed as significantly different connectivities in Figures 3, 4, the characteristic parameters based on graph theory analysis of the brain functional network were calculated and used as features in machine learning by decision trees. As shown in the previous results, when classifying FESZ, UHR, and HC, the classifier that adds neurophysiological characteristics (i.e., P50 performance and brain functional network parameters) to MCCB and demographic characteristics provides improved accuracy. Besides the results given in Table 4, four classifiers by which the four types of features were separately practiced. The results show that the accuracy of the brain network parameters was lower than that of MCCB, which had the highest accuracy. However, the accuracy of each of the four features was much lower than that obtained by the integration of the four features. Taken together, when brain functional network parameters based on P50 suppression are combined with other features, such as cognitive features measured by MCCB, it can provide more evidence for early prediction of schizophrenia.

## Conclusion

Taken together, the results of this study indicate that brain functional network based on EEG of P50 paradigm may be helpful for the identification of different stages (FESZ, UHR, HC) of schizophrenia and assist early diagnosis of schizophrenia. In addition, there was almost no significant difference between UHR and FESZ in the gating response (i.e., the S1–S2 period); however, the gating response between each of these groups (UHR, FESZ) and HC was significantly different; FESZ had a stronger connection in the right superior frontal gyrus and right insula than HC; UHR had an enhanced connection in the paracentral lobule and middle temporal gyrus compared with HC. The difference between UHR and FESZ was more at the baseline (i.e., the S1 period). Moreover, brain functional network parameters (including clustering coefficient, characteristic path length, and global efficiency) extracted based on graph theory improved the accuracy of the three-group classifier for FESZ, UHR, and HC. The present study characterizes the relationship between P50 suppression and different clinical stages of schizophrenia, and thus enhances our understanding of mechanism of P50 suppression in schizophrenia. And it provides further evidence for potentially useful applications of P50 suppression together with cognitive assessment and clinical examinations in the diagnosis and possible early intervention of schizophrenia.

## Data Availability Statement

The datasets generated for this study are available on request to the corresponding author.

## Ethics Statement

The studies involving humans were reviewed and approved by the Ethics Committee of Beijing Anding Hospital. Written informed consent was obtained either from the participants or from the participants’ legal guardian/next of kin where required.

## Author Contributions

QC contributed to data processing, analysis of results, and writing of manuscript. QT was responsible for the design of the experiment and the collection of data. ML, HW, and YL revised the manuscript and carried out literature research. CW and JZ was in charge of the design and implementation of the experiment, as well as the interpretation of the results.

## Conflict of Interest

The authors declare that the research was conducted in the absence of any commercial or financial relationships that could be construed as a potential conflict of interest.

## Footnotes

1 https://www.mathworks.com/products/matlab.html

2 https://sccn.ucsd.edu/eeglab/index.php

3 https://www.uzh.ch/keyinst/loreta
