# Supplementary material for: EEG-Based Brain Functional Connectivity in First-Episode Schizophrenia Patients, Ultra-High-Risk Individuals, and Healthy Controls During P50 Suppression
Source: Front Hum Neurosci. 2019 Nov 14;13:379. doi: 10.3389/fnhum.2019.00379 (PMC6870009; doi:10.3389/fnhum.2019.00379)
Supplement: Supplementary file 1 [file Data_Sheet_1.docx]

Supplementary Material

# Supplementary Table

**Supplementary Table 1.** Coordinates of the 80 cortical points corresponding to AAL atlas used for connectivity analyses.

|  | Left hemisphere | | | Right hemisphere | | |
| --- | --- | --- | --- | --- | --- | --- |
|  | x | y | z | x | y | z |
| Media orbitofrontal cortex | -5 | 55 | -5 | 5 | 50 | -5 |
| Middle orbitofrontal cortex | -30 | 50 | -10 | 30 | 55 | -10 |
| Superior frontal gyrus, medial part | -5 | 50 | 30 | 10 | 50 | 30 |
| Superior frontal gyrus, orbital part | -20 | 50 | -15 | 15 | 50 | -15 |
| Anterior cingulate cortex | -5 | 35 | 15 | 5 | 35 | 15 |
| Middle frontal gyrus | -35 | 35 | 35 | 35 | 35 | 35 |
| Superior frontal gyrus | -20 | 35 | 40 | 20 | 30 | 45 |
| Gyrus rectus | -5 | 35 | -20 | 5 | 35 | -20 |
| Inferior frontal gyrus, orbital part | -35 | 30 | -10 | 40 | 30 | -10 |
| Inferior frontal gyrus, parts triangularis | -45 | 30 | 15 | 45 | 30 | 15 |
| Inferior frontal operculum | -50 | 15 | 20 | 50 | 15 | 20 |
| Olfactory gyrus | -5 | 15 | -10 | 5 | 15 | -10 |
| Temporal pole, middle temporal gyrus | -35 | 15 | -35 | 45 | 15 | -30 |
| Temporal pole, superior temporal gyrus | -40 | 15 | -20 | 45 | 15 | -15 |
| Insula | -40 | 10 | 0 | 40 | 10 | 0 |
| Supplementary motor area | -5 | 5 | 60 | 10 | 0 | 60 |
| Precentral gyrus | -40 | -5 | 50 | 40 | -10 | 50 |
| Rolandic operculum | -50 | -10 | 15 | 50 | -5 | 15 |
| Middle cingulate cortex | -5 | -15 | 40 | 5 | -10 | 40 |
| Parahippocampal gyrus | -20 | -15 | -20 | 20 | -15 | -20 |
| Heschl gyrus | -45 | -20 | 10 | 45 | -15 | 10 |
| Hippocampus | -25 | -20 | -10 | 25 | -20 | -10 |
| Superior temporal gyrus | -55 | -20 | 5 | 55 | -20 | 5 |
| Paracentral lobule | -5 | -25 | 70 | 5 | -30 | 70 |
| Postcentral gyrus | -45 | -25 | 50 | 40 | -25 | 55 |
| Inferior temporal gyrus | -50 | -30 | -25 | 55 | -30 | -20 |
| Supramarginal gyrus | -55 | -35 | 30 | 55 | -30 | 35 |
| Middle temporal gyrus | -55 | -35 | 0 | 55 | -35 | 0 |
| Fusiform gyrus | -30 | -40 | -20 | 35 | -40 | -20 |
| Posterior cingulate cortex | -5 | -45 | 25 | 5 | -45 | 20 |
| Inferior parietal lobule | -45 | -45 | 45 | 45 | -45 | 50 |
| Precuneus | -10 | -55 | 50 | 10 | -55 | 45 |
| Superior parietal lobule | -25 | -60 | 60 | 25 | -60 | 60 |
| Angular gyrus | -45 | -65 | 40 | 40 | -60 | 40 |
| Lingual gyrus | -15 | -70 | -5 | 15 | -65 | -5 |
| Calcarine sulcus | -10 | -80 | 10 | 15 | -75 | 10 |
| Cuneus | -5 | -80 | 25 | 15 | -80 | 30 |
| Inferior occipital gyrus | -35 | -80 | -10 | 35 | -80 | -10 |
| Middle occipital gyrus | -30 | -80 | 15 | 35 | -85 | 20 |
| Superior occipital gyrus | -20 | -85 | 30 | 20 | -80 | 30 |

# Supplementary Figure


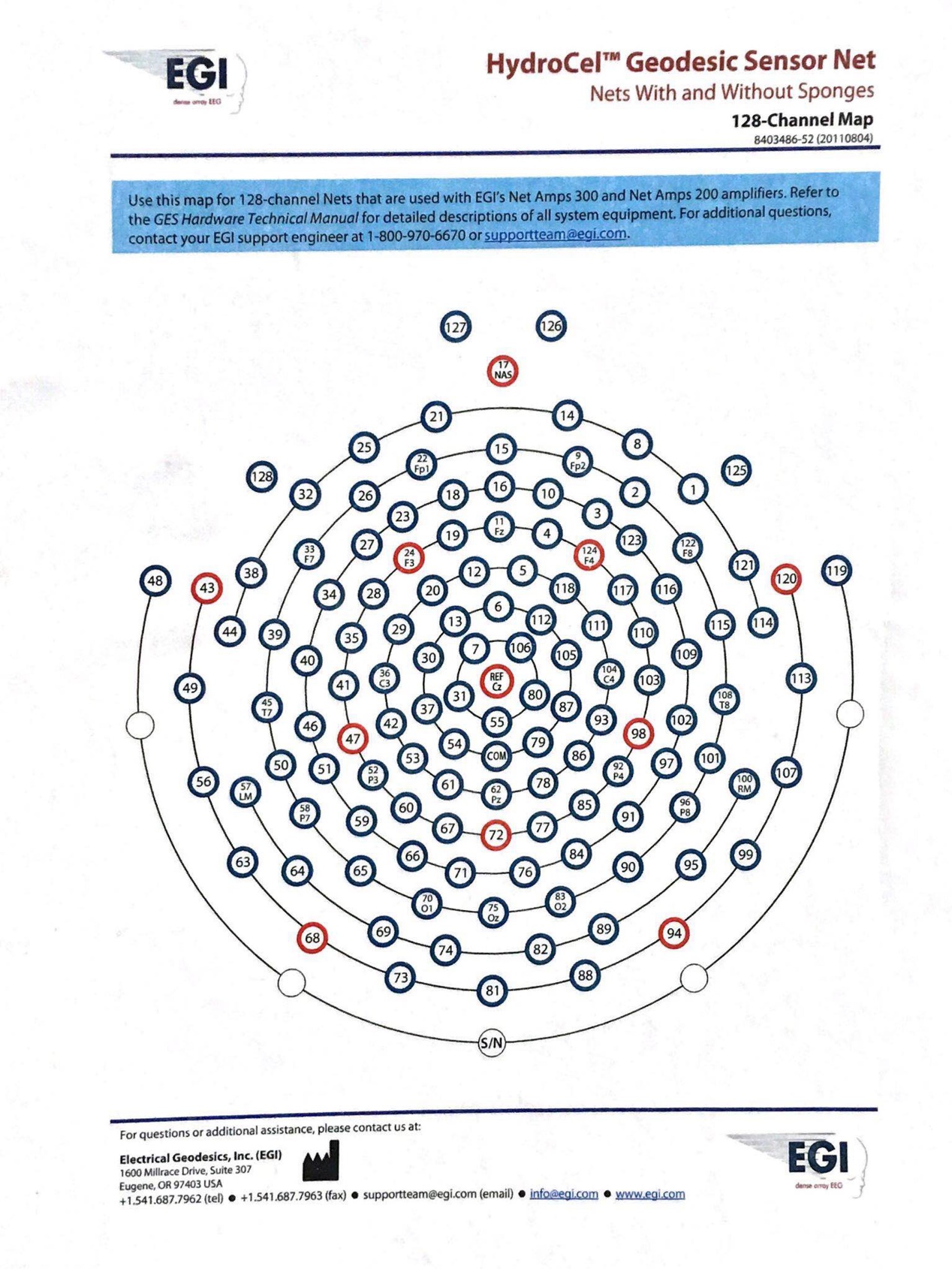


**Supplementary Figure 1.** The distribution of all 128 electrodes during EEG recording.
